# Life-Threatening Mediastinal Hematoma Formation After Removal of the Hemodialysis Catheter in a Boxer: A Case Report

**DOI:** 10.3389/fvets.2021.691472

**Published:** 2021-07-05

**Authors:** Athanasia Mitropoulou, Hendrik Lehmann, Evelyn M. Heier, Matthias Schneider, Esther Hassdenteufel

**Affiliations:** Department of Veterinary Clinical Sciences, Small Animal Clinic, Justus-Liebig-University Giessen, Giessen, Germany

**Keywords:** mediastinal hematoma, hemodialysis catheter complications, central venous catheter (CVC), low molecular weight heparin (LMWH), antifactor Xa, acute kidney injury, continuous renal replacement therapy, canine (dog)

## Abstract

A 4-year-old female Boxer was referred for renal replacement therapy 2 days after observed grape ingestion. An 11-French dual-lumen dialysis catheter was placed into the right jugular vein and continuous renal replacement therapy was initiated for 66 h. Afterwards the patient received enoxaparin subcutaneously as a thromboprophylaxis. Four hours after removal of the dialysis catheter the patient developed severe dyspnea with hypercapnia and signs of hemorrhagic shock. Bedside ultrasound and X-rays of the thorax revealed a soft tissue opacity dorsally of the trachea, located in the mediastinum. The findings were consistent with mediastinal bleeding and hematoma formation. Blood gas examination indicated hypoventilation. The dog was managed conservatively with multiple blood transfusions and mechanical ventilation. The patient survived to discharge, and the hematoma was fully absorbed in the radiographs after 17 days. Patients with impaired kidney function should receive individualized enoxaparin dosage adjusted to anti-Xa levels and should be strictly monitored for complications. Mediastinal hemorrhage and hematoma formation should be considered as a potential complication in patients receiving a jugular vein catheter.

## Introduction

For successful delivery of extracorporeal renal replacement therapy, vascular access is crucial. In veterinary medicine, vascular access for continuous or intermittent hemodialysis (HD) is usually provided by a temporary, dual-lumen catheter placed percutaneously in the jugular vein (1). The most common complications seen with HD catheters are catheter dysfunction due to malposition or mechanical kinking of the catheter, catheter-related thrombosis and bacterial infections (2–5). A rare non-infectious complication of HD catheters in human medicine is vascular erosion following the guidewire's insertion or the catheter. Vascular erosion can lead to severe complications such as hemothorax, hemomediastinum, or pericardial tamponade (6, 7). These complications are often fatal due to delayed diagnosis. In the veterinary literature, hemomediastinum as complication of HD catheters has not been reported yet. This is the first report of mediastinal hematoma formation in a dog after removal of the HD catheter.

## Case Summary

A 4-year-old, 26 kg, female Boxer, was referred to a veterinary teaching hospital for renal replacement therapy with an oliguric acute kidney failure due to ingestion of grapes. At presentation, the patient was dull, arrhythmic and showed extreme salivation. Further vital parameters were within normal limits, the urinary bladder was small. The initial blood work revealed a thirteen-times elevation of creatinine, a moderate potassium elevation, a severely low ionized calcium, as well, as a mild hypercoagulability assessed by kaolin-activated thromboelastography (TEG® 5000, Haemonetics Inc., Braintree MA, USA) (Table 1). The initiated medical treatment with infusion of 0,9% NaCl (B.Braun Melsungen AG, DE), furosemide bolus and continuous rate infusion (CRI) (Dimazon 50 mg/ml, Intervet, Unterschleißheim, DE), 10% calcium gluconate (Calciumgluconat 10% B. Braun) and mannitol (Osmofundin 15%, B. Braun Melsungen AG, DE) resulted in a temporary decrease in serum potassium concentration but did not increase urine output. According to the International Renal Interest Society (IRIS) Guidelines (9), the patient was graded as Acute Kidney Injury (AKI) Grade 5 oliguric and in need of renal replacement therapy.

**Table 1:** Summary of the blood results at presentation.

**Parameter**	**Day 1**	**Day 6 (8 pm)**	**Day 7 (5 am)**	Day 7^**^ (8–10 am)	**Reference range**
WBC	10.13	22.94			5.05 – 16.76 ×10^9^/l
Neutrophils	8.6	19.63			2.95 – 11.64 ×10^9^/l
Lymphocytes	0.97	1.71			1.05 – 5.1 ×10^9^/l
Monocytes	0.5	1.57			0.16 – 1.12 ×10^9^/l
Eosinophils	0.05	0.00			0.06 – 1.23 ×10^9^/l
Basophils	0.01	0.03			0 – 0.1 ×10^9^/l
Hematocrit	30.6	16			37.3 – 61.7%
RBC	5.44	2.84			5.65 – 8.87 ×10^12^/l
Reticulocytes	6.5	38.6			10 – 110 10^9^/l
PLT	316	162			148 – 484 ×10^9^/l
CRP	10.6			58.4	0 – 14.9 mg/dl
Urea	45.7			18.4	3.3 – 9.82 mmol/l
Creatinine	1,462			241	53 – 122 μmol/l
Sodium (Na)	146			142	142 – 149 mmol/l
Potassium (K)	7.04			3.61	3.35 – 4.37 mmol/l
Ionized Calcium (iCa)	0.62			1.19	1.23 – 1.43 mmol/l
Phosphor	4.59			2.44	0.79 – 2.1 mmol/l
Albumin	23.4			21.1	29.6 – 37.01 g/l
Globulin	34.8			26	22.9 – 35.6 g/l
Total Solids	58.2			47.1	55.3 – 69.8 g/l
Glucose	5.9			6.5	3.3 – 6.53 mmol/l
Bilirubin	3.08			5.73	0 – 3.6 μmol/l
Cholesterol	6			5.6	3.3 – 8.6
Triglycerides	0.19			0.7	0.08 – 0.75
Alkaline phosphatase (ALP)	138			77	0 – 130 U/l
Alanine-amino-transferase (ALT)	108			73	0 – 85 U/l
Glutamatdehydrogenase (GLDH)	10			1	0 – 9.9 U/l
Gamma-glutamyltransferase (g-GT)	15			8	<6.4 U/l
DGGR Lipase	661			404	<300 U/l
pH (venous)	7.386	7.315	7.182		7.35 – 7.45
pvCO_2_	41.5	60	84.7		35 – 45 mmHg
Bicarbonate	24.3	29.9	31		19 – 25 mmol/l
Base excess (BE)	−0.7	3.3	1.6		−5 – 5 mmol/l
Lactate	1.8	2.9	0.8		0.4 – 2.2 mmol/l
Activated Clotting Time (ACT)	Double measurement:	Double measurement:			66.5 – 97.0 s
	102/104	128/130			
Fibrinogen	2.41			6.77	1.1 – 3.5 g/l
D-Dimers				0.37	<0.67
Anti-thrombin III				82	107 – 128%
Prothrombin time (PT)				8.5	6.52 – 8.16 s
Activated partial thromboplastin time (APTT)				13.2	9.85 – 14.22 s
**Kaolin activated thromboelastography (TEG)**					
**Parameter**					Reference range^*^
Reaction time (R)	3.7			4.3	2 – 9 min
Clot formation time (K)	0.8			1.2	1 – 6 min
Angle (a)	77.2			72.8	37 – 75°
Maximal Amplitude (MA)	63.4			68.8	43 – 68 mm
G – value (G)	8.6 K			11 K	3.2 – 9.6 K (d/sc)

*Hematology (ProCyte Dx, Hematology Analyzer, IDEXX Europe, B.V, Hoofddorp, NE), biochemistry panel (ABX Pentra C400, Horiba ABX SAS, Montpellier), blood gas analysis (cobas b 221 POC system, Roche Diagnostics GmbH, Mannheim), and the following coagulation parameters: activated clotting time (ACT) (Hemotec Medronic ACT II Coagulation Analyzer, Soma Technology, Bloomfield, USA), fibrinogen (STA Compact MAX3 –Stago Deutschland GmbH, Duesseldorf, DE) and thromboelastography (TEG® 5000,Haemonetics Inc., Braintree MA, USA) are presented. The boxes are colored with green for normal value, blue for decreased value, red for increased value, and gray for missing value*.

* *Bauer et al. (8)*.

** *Coagulation assessment on day 7 in the morning was performed after transfusion therapy with two FFPs and one pRBC and after treatment with tranexamic acid*.

Under mild sedation with butorphanol (0.3 mg/kg iv, Butomidor 10 mg/kg inj.,Vetoquinol, Ismaning, DE) and without any difficulties, an eleven-French (Fr.) 20 cm temporary dual-lumen dialysis catheter (Proven Care Catheter Sets, Fresenius, Bad Homburg, DE) was placed into the right jugular vein using the modified percutaneous Seldinger technique (1). Continuous renal replacement therapy (CRRT) (multiFiltrate Ci-Ca CVVHD, Fresenius Medical Care, Bad Homburg, DE) with regional citrate anticoagulation was initiated for 66 h. As there is no integrated monitoring system in the dialysis machine, the circuit post-filter iCa is measured after 5 min and after 1 h of treatment start with a blood gas analysis machine (cobas b 221 POC system, Roche Diagnostics GmbH, Mannheim). The targeted circuit post-filter iCa is 0.25 – 0.34 mmol/l. If the citrate flow needs to be adjusted the post-filter iCa should be checked again after 1 h. If not, the circuit post-filter iCa should be monitored every 3–6 h. The systemic (patient's) iCa should be monitored every 8 h with a targeted range of 1.05 – 1.20 mmol/l. Additionally, a continuous rate infusion (CRI) of unfractionated heparin (Heparin-Natrium 25.000 I.E./5 ml, B.Braun, Melsungen, DE) was used (10) adjusted to achieve an activated clotting time (ACT) (Hemotec Medronic ACT II Coagulation Analyzer, Soma Technology, Bloomfield, USA) of 130–150 s. As the patient had no tendency to bleed, she received directly prior of starting the CRRT a heparin bolus (50 U/kg) to a target of a pre-dialysis ACT of > 200. Moreover, the extracorporeal system was primed with heparin as the CVVHD machine has an integrated heparin pump. The systemic bolus of heparin is used to avoid overzealous coagulation in the circuit if there are problems or frequent interruptions during the start of the dialysis process. The heparin CRI was used as additional low-dose heparinization during the days of the dialysis, also to avoid clotting in the patient at the large catheter in the jugular vein. For the 66 h of CRRT the ACT was kept in the desired range using a dosage of heparin CRI between 12.5 and 45 U/kg/h. The patient tolerated the CRRT well, no treatment interactions due to catheter related problems or clots were noted. After 66 h, the CRRT was discontinued due to clinical improvement and increased urinary output. Directly afterwards the ACT of the patient was 125/111. The jugular catheter remained in place, until the patient reached a stable clinical and metabolic state. The 11 Fr. catheter used has a volume of 1.45 ml in the venous (distal) port and 1.4 ml in the arterial (proximal) port. In order to prevent any thrombus formation in the lumen, both ports were locked with a solution of heparin mixed with NaCl resulting in a concentration of 2,500 U/ml heparin. Twelve hours after stopping CRRT and thus the heparin CRI, a dose of 1 mg/kg enoxaparin was administrated with an insulin syringe [needle size 0.30 mm (30G) ×8 mm] subcutaneously (s.q.) every 8 h, (Clexane 100 mg multidose, Sanofi-Aventis, Frankfurt am Main, DE) as a general thromboprophylaxis. Before this case, this used to be a common practice in our institution in patients when the primary disease had no increased bleeding risk as large CVCs result in activation of coagulation potentially leading to jugular vein thrombosis. Many of these patients have co-existing diseases (e.g., pancreatitis) that might lead to an additional hypercoagulable state. The removal of the catheter was performed 2 days after the end of the CRRT and 7 h after the last s.q. administration of enoxaparin and after aspiration of the heparin-lock solution from both catheter ports. Pressure was applied for 5 min, and the neck was bandaged. One hour after removal the dog received the next (fifth) s.q. injection of enoxaparin, as planned in the rhythm of three times daily (TID) injections.

**Figure 1:**
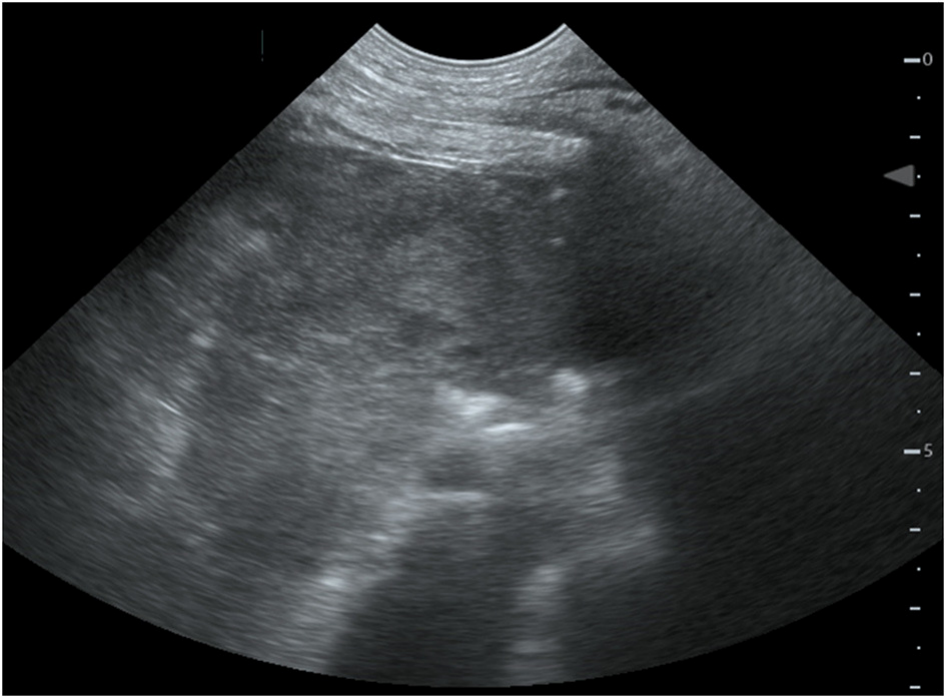
Bedside ultrasound of the thorax. Left paracostal view of a 4-year old female Boxer with mediastinal hematoma. Located dorsally to the heart, a hyperechoic well-demarcated intrathoracic structure is pictured.

**Figure 2:**
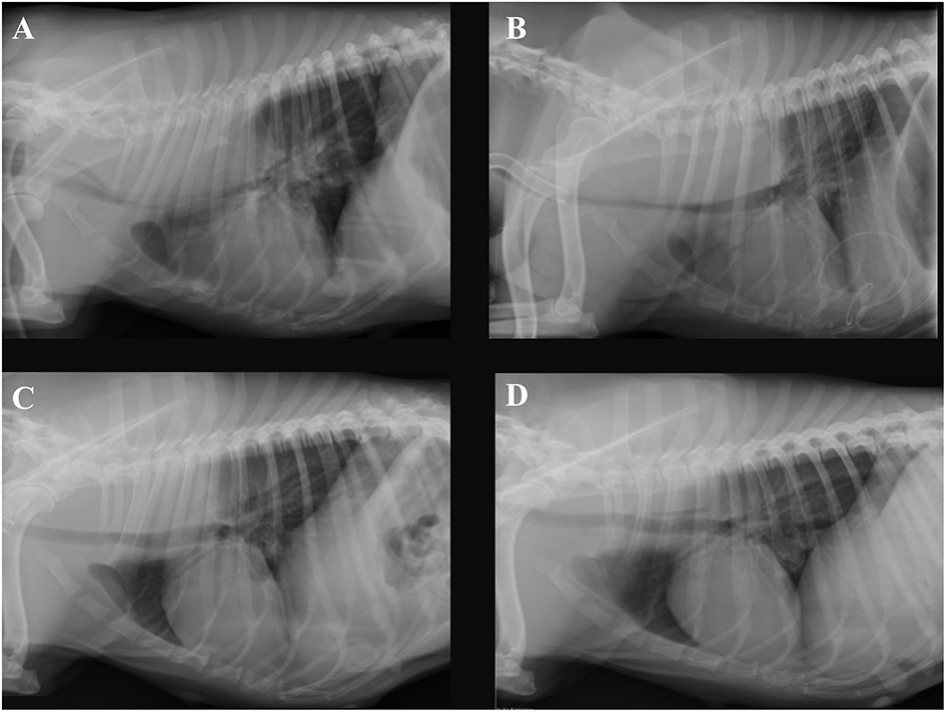
Right lateral thoracic radiographs of the same dog. **(A)** Radiograph obtained on day 6 of hospitalization, 4 h after removing the dialysis catheter, **(B)** obtained on day 11, **(C)** on day 16, and **(D)** on day 23. A soft tissue mass dorsally of the trachea inside the mediastinum is present. Progressive shrinking of the mass to its disappearance through the days of hospitalization can be seen.

Four hours after removing the catheter, the dog deteriorated rapidly. She developed severe mixed dyspnea, her mucous membranes became pale, and she was tachycardic (160/min) with a weak pulse and a muffled pulmonary and heart auscultation ventrally of the thorax. The first blood analysis revealed a moderate hypercapnia and a severe anemia as seen on Table 1. The ACT at this time was only mildly elevated with 128 and 130 s (11). The clinical picture and laboratory values were consistent with acute hemorrhage, hypoventilation and hypovolemic shock. The bedside ultrasound (TE7 ACE, Mindray Medical Germany GmbH, Darmstadt, DE) of the chest (Figure 1) revealed moderate hypovolemia as well as a hyperechoic well-demarcated intrathoracic structure, located dorsally of the heart. Moreover, a mild amount of pleural effusion was detected. The main findings of the chest radiographs (Figure 2A) were a soft tissue opacity dorsally of the trachea, located in the mediastinum, which deviated the trachea, the esophagus, the pulmonary vessels, and the heart silhouette ventrally. The lung parenchyma of the right cranial and middle lobe, as well as the left cranial lobe, showed an alveolar pattern and lobar signs. The findings were consistent with mediastinal bleeding and hematoma formation, as well as compression of the main bronchi. The observed lung pattern could have been due to a combination of atelectasis and non-cardiogenic lung edema because of airway obstruction from the hematoma.

**Figure 3:**
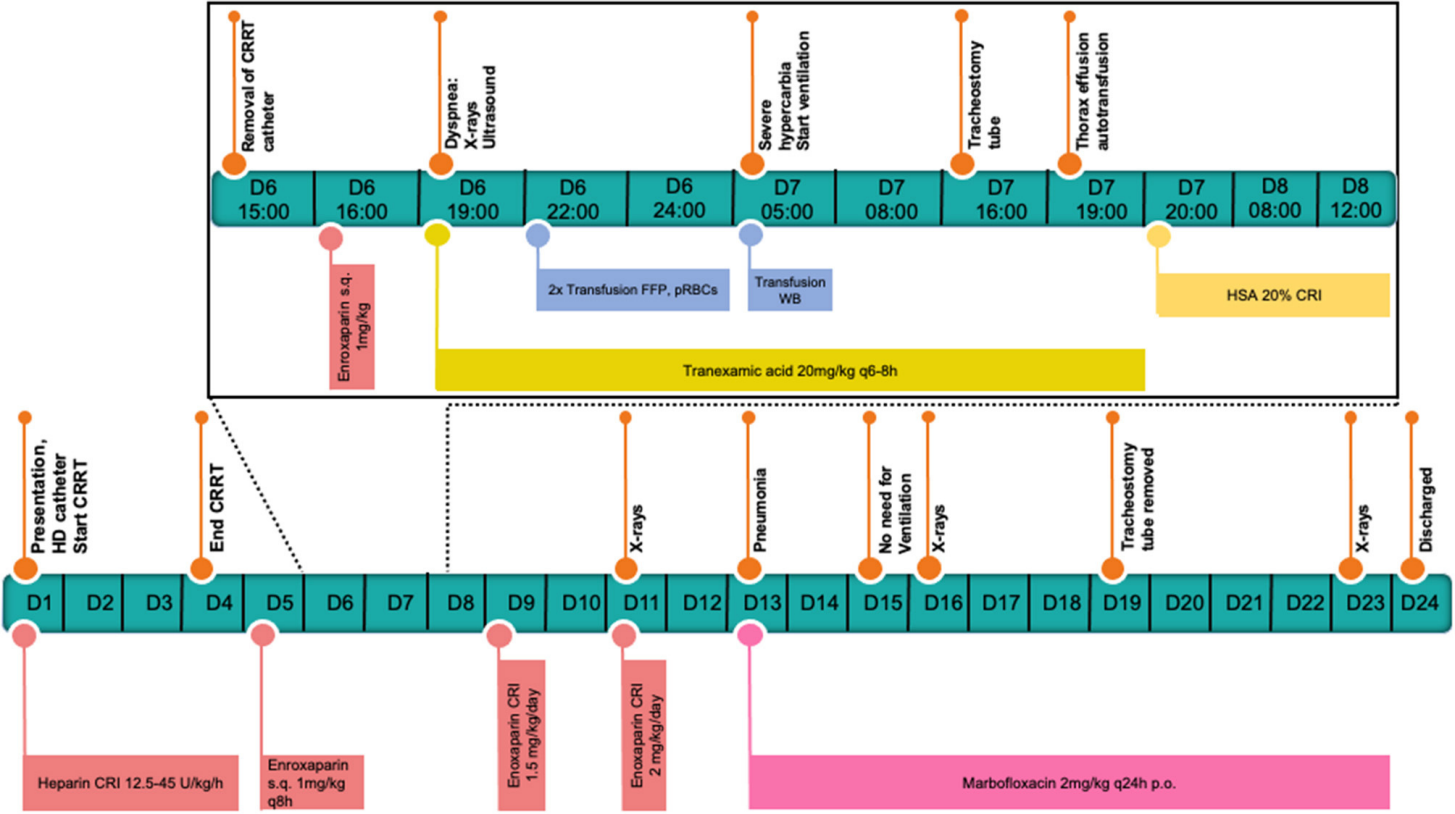
Schematic presentation of the case management. The critical 6th day until the 8th day are presented hourly including the stabilization measurements.

In the next 12 h after the deterioration, the patient was stabilized with a balanced electrolyte solution (Sterofundin® ISO, B.Braun, Melsungen AG, DE) for treatment of hypovolemia as needed, tranexamic acid (20 mg/kg every 6–8 h,Cyklokapron®, Pfizer, Puurs, BE), two units of fresh frozen plasma (FFP) as well as packed red blood cells (15 ml/kg pRBCs). Also, a chest tube was placed, and the thorax was drained. Approximately 12 h after catheter removal, the breathing pattern of the patient was further deteriorating, and her neck and head started to swell up. Worsening of the hypercapnia resulted in severe respiratory acidosis (see Table 1) and the decision was made to induce general anesthesia (premedication with midazolam 0.2 mg/kg iv, Midazolam-hameln 5 mg/ml, Hameln, DE, induction with propofol 4 mg/kg iv, Propofol Hexal 10 mg/ml, Holzkirchen, DE, maintenance with fentanyl CRI 2.5 – 5μq/kg/h, Fentadon, Dechra Veterinary Products, Aulendorf, DE, midazolam CRI: 0.1 – 0.2 mg/kg/h and later pentobarbital CRI 0.2 – 2 mg/kg/h, Narcoren 16 g/100 ml, Vetmedica GmbH, Ingelheim, DE, for a total of 14 h) and ventilate (Siemens Maquet SERVO – S, Rastatt, DE) with pressure-controlled ventilation. The patient also received a surgically placed tracheostomy tube for the time she remained on the ventilator. The same morning that the mechanical ventilation was started, further stabilization of the hematocrit with one unit of whole blood (16 ml/kg WB) from donors of the same blood type after compatible cross-matching was performed. Over these first 12 h, 1,300 ml of pleural effusion were drained. The fluid was serosanguineous with a PCV of 5% and a relatively low total nucleated cell count (TNCC 2.64 ×10^3^μl). A total of 150 ml was reinfused after washing and processing with an autotransfusion device (OrthoPat, Haemonetics, Braintree MA, USA). The head and neck swelling further worsened, and moderate hypoalbuminemia (21.1 g/l, range: 29.6 – 37.01 g/l) was detected. As an attempt to increase oncotic pressure and to decrease the edema formation, a total of 40 g of human serum albumin 20% (CSL Behring GmbH, Marburg, DE; 0.5 ml/kg/h over 16 h) was administrated. One day after these stabilization measures, clinical and laboratory values improved. At the echocardiography, a mild amount of thrombotic material was suspected in the right atrium. Two days after the incident and to prevent further thrombus formation, and enoxaparin CRI was started, initially at a low dose of 1.5 mg/kg/day. Anti-factor Xa was measured regularly to avoid overzealous anticoagulation and prevent further bleeding. The values with that dosage after 24 and 48 h were 0.19 and 0.47 IU, which lie below the recommended reference range for thromboprophylaxis in the literature (0.5 – 1.0) (12). The following days the dog remained under mild sedation (fentanyl CRI: 2.5 – 5 μg/kg/h for 1 day, and the next 5 days with butorphanol CRI: 0.1 – 0.2 mg/kg/h, and if needed acepromazine: 0.01 – 0.02 mg/kg iv, Prequilan 10 mg/ml, Fatro SpA, IT) on the ventilator mainly on PSV mode (Pressure Support Ventilation) alternating with CPAP mode (Continuous Positive Airway Pressure). This support seemed to be necessary to overcome the tracheal compression induced by the mediastinal hematoma formation. The duration of time without ventilator support could be increased from day to day. On the 7th day on the mechanical ventilator, the inflammation parameters in the blood increased, the aspirate out of the endotracheal tube suction became purulent, and the patient's oxygenation levels dropped slightly. The development of ventilator-associated pneumonia was strongly suspected. Blood and tracheal aspirate cultures were taken and revealed growth of multi-resistant bacteria (*E.coli and A.baumanii*). The patient was already receiving amoxicillin-clavulanic acid, 20 mg/kg iv every 8 h, (AmoxClav Hexal 500/100 mg, Hexal, Holzkirchen, DE) as one concern was that leptospirosis could also be responsible for the acute kidney failure. So, another antibiotic drug, marbofloxacin, 2 mg/kg iv and later orally, Marbocyl FD 1%, Vetoquinol, Ismaning, DE, was administered when symptoms started. Clinical improvement of the patient and decrease of the inflammatory markers could be observed. Further cultures of blood and urine before discharge yielded no bacterial growth. The dog was weaned from the ventilator after 8 days. The tracheostomy tube was removed after 12 days. The progression of the hematoma shrinking to its disappearance is visualized in Figures 2B–D. An echocardiography on the 21st day of hospitalization revealed no thrombotic material. A schematic presentation of the case management can be seen in Figure 3. The kidney values were within the normal range at the time of hospital discharge and remained normal until today (2 years after presentation). Three months after discharge the urine specific gravity was 1.026, the blood pressure and the SDMA concentration were slightly elevated (SDMA: 16 μg/dl, range: 0 – 14 μg/dl) and the dog was staged at that time as IRIS Stage 1 borderline hypertensive, not proteinuric. Nine months after discharge, the SDMA concentration was normal (14 μg/dl).

## Discussion

This case describes the formation of a mediastinal hematoma after removal of a hemodialysis catheter. The diagnosis was based on clinical presentation, laboratory values, as well as on radiographic and sonographic imaging of the thorax. To the knowledge of the authors, this potentially fatal complication has not been described before in the veterinary literature. Furthermore, this case report provides information about the management of such patients. It also reports the duration of a hematoma of that size until its reabsorption, with a long term follow up period (24 months) with a successful outcome.

Mediastinal hemorrhage and hematomas are relatively uncommon in dogs, and it is mostly seen as a sequel of anticoagulant rodenticide toxicity. Three studies reported hemomediastinum due to anticoagulant rodenticide toxicity, with one of them being in juvenile dogs due to thymic hematoma (13–15). Besides an impaired coagulation system, mediastinal hematomas can be a result of trauma or bleeding mediastinal neoplasia (16, 17).

On the other hand, several reports of mediastinal hemorrhage and hematoma formation caused by vascular erosion during the insertion of CVC or due to malposition of CVC exist in the human literature. Specifically, a total of 13 cases of female and male adult patients were found (18–30). In these reports, due to clinical deterioration of the patients from shortly after, until 4 days after insertion of the CVC, further investigations were performed, and mediastinal hematoma was diagnosed. Of all the patients, four had a surgical approach after diagnosis with traditional exploratory sternotomy in three and video-assisted thoracotomy in one patient (18, 20, 22, 24). All but one of the patients that received surgery survived. The one patient that died, required surgical vascular repair and was the one where the diagnosis was delayed for 4 days (18). All remaining nine out of the 13 cases that were treated with conservative management survived, and three of them required ventilation (19, 23, 25).

There are no reports in the human literature of a mediastinal hemorrhage and hematoma formation after removing a CVC catheter. Considering the course of the events in this case, it is most likely that the post-dialysis heparinization treatment resulted in the reported events after the CVC removal. The prolonged clotting time measured at the point of the deterioration indicates that the patient had an impaired coagulation possibly due to the use of enoxaparin. Moreover, it can be suggested that while inserting the CVC before the HD, a vascular erosion occurred. However, initially, the dog was not under heparin treatment and 2 h elapsed from the insertion of the catheter until the heparin treatment started, enough time for a vascular plug to form. Because of the slower blood flow of the CRRT and the relatively low dosage of heparin, no problems did occur while on CRRT. The removal of the CVC probably resulted in simultaneous removal of the previously formed vascular plug, leading to a new vascular erosion. At that time, the patient was receiving thromboprophylaxis, leading to ongoing bleeding and hemomediastinum. There are many thromboprophylaxis protocols for patients with hemodialysis catheters in place with more common the one of heparin locks at the catheter ports combined with an oral anti-platelet agent such as clopidogrel or aspirin. In our institution we tend to avoid platelet aggregation inhibitors in patients with a bleeding risk or planned invasive procedure for some reasons. Firstly, the duration of action is prolonged, after discontinuing the drug it takes around 5–7 days to regain normal platelet function. Secondly, the monitoring assays (platelet aggregometry) are not routinely available for clinical use whereas the anti-factor Xa assay is available through the main laboratory. As enoxaparin is administrated subcutaneously with an insulin syringe, bleeding complications from the injection site did not occur.

Low molecular weight heparins (LMWHs) are primarily eliminated via the kidneys. Given that, the 2012 CHEST guidelines on antithrombotic therapy in humans raise concerns about the accumulation of the drug and the bleeding risks in patients with renal impairment (31). In the human literature, enoxaparin clearance was reduced by 17–44% in patients with mild to moderate renal dysfunction (32), and thus, several studies suggest an individualized LMWH dosage using anti-Xa levels as a therapeutic target (33–36). Regarding the use of thromboprophylaxis in patients with acute kidney disease in veterinary medicine, there are no studies to support its use. The CURATIVE Guidelines define dogs with protein-losing nephropathy as a risk population and support the use of antithrombotic therapy (37). It is documented that dogs with AKI have an impaired primary hemostatic function (38). Francey et al. evaluated the hemostatic function of 256 dogs with renal disease and concluded that dogs with AKI had the broadest spectrum of hemostatic disorders, with 15% being hypercoagulable and 79% having mixed hypo-hypercoagulable features (39). Regarding our case, it is a limitation that neither extended hemostasis assessment after CRRT and before starting LMWH, nor reduced enoxaparin dosage was considered. At the point of clinical deterioration anti-Xa levels should have been measured. However, the initial TEG of the patient was mild hypercoagulable and at night no extended laboratory testing was possible. The use of protamine sulfate as a reversal agent of LMWH activity could have been considered in this case (40). However, the dosage is unclear, anaphylactic response may occur and studies about its usage in dogs are lacking. The same, including the unbearable financial cost, applies for andexanet alpha a recombinant modified human FXa protein that can be used not only as a neutralizer for the newer direct oral FXa inhibitors (apixaban and rivaroxaban) but also for the anti-thrombin dependent indirect inhibitors such as enoxaparin (41, 42).

The use of human serum albumin (HSA) in veterinary critically ill patients remains controversial. The decision on this particular case was based upon the clinical deterioration of the patient with excessive head and neck swelling despite previous administration of multiple FFP und WB units, as well as, after careful consideration of the side effects and discussion with the owner. The dog did not show any acute or delayed reactions after the HSA administration, and the kidney function was not influenced. It should be pointed out, that the albumin concentration of the patient was not low enough to explain the excessive swelling of the head and neck. It is possible that the hematoma caused a compression of the main vessels, especially the vena cava cranialis.

Finally, computed tomography (CT) scans of the thorax at the night of the deterioration could have been performed to examine any ongoing bleeding that would need surgical repair. We decided not to proceed with CT scans initially due to patient instability as well as restrained use of contrast medium due to impaired kidney function. The next day when the dog was stable enough to proceed to CT scans, the owner rejected it for financial reasons.

## Concluding Remarks

This case report describes a life-threatening hematoma in a dog receiving LMWH after removing the HD catheter. It was managed conservatively, remained on the ventilator for 8 days, and the hematoma was fully reabsorbed after 17 days. Patients with impaired kidney function should receive individualized LMWH dosage in respect of anti-Xa levels and should be strictly monitored for complications. Mediastinal hemorrhage and hematoma formation should be considered as a potential complication in patients receiving a jugular vein catheter.

## Data Availability Statement

The original contributions presented in the study are included in the article/supplementary material, further inquiries can be directed to the corresponding author/s.

## Ethics Statement

The dog detailed in this case report presented as a patient to the Department of Veterinary Clinical Sciences, Small Animal Clinic, Justus-Liebig-University Giessen in Giessen, Germany. The owners signed a consent to permit hospitalization and treatment. Informed consent was obtained from the owner prior to the writing and submission of this case report.

## Author Contributions

AM assisted in primary case management and wrote the manuscript. HL and EMH assisted in the case management. EH and MS supervised the clinical management of the case. All authors critically reviewed and approved the final version of the manuscript.

## Conflict of Interest

The authors declare that the research was conducted in the absence of any commercial or financial relationships that could be construed as a potential conflict of interest.
